# Idiosyncratic biases in the perception of medical images

**DOI:** 10.3389/fpsyg.2022.1049831

**Published:** 2022-12-19

**Authors:** Zixuan Wang, Mauro Manassi, Zhihang Ren, Cristina Ghirardo, Teresa Canas-Bajo, Yuki Murai, Min Zhou, David Whitney

**Affiliations:** ^1^Department of Psychology, University of California, Berkeley, Berkeley, CA, United States; ^2^School of Psychology, University of Aberdeen, King’s College, Aberdeen, United Kingdom; ^3^Vision Science Group, University of California, Berkeley, Berkeley, CA, United States; ^4^Center for Information and Neural Networks, National Institute of Information and Communications Technology, Koganei, Japan; ^5^Department of Pediatrics, The First People's Hospital of Shuangliu District, Chengdu, Sichuan, China; ^6^Helen Wills Neuroscience Institute, University of California, Berkeley, Berkeley, CA, United States

**Keywords:** medical image perception, individual differences, GAN-simulated medical images, perceptual biases, radiologist performance

## Abstract

**Introduction:**

Radiologists routinely make life-altering decisions. Optimizing these decisions has been an important goal for many years and has prompted a great deal of research on the basic perceptual mechanisms that underlie radiologists’ decisions. Previous studies have found that there are substantial individual differences in radiologists’ diagnostic performance (e.g., sensitivity) due to experience, training, or search strategies. In addition to variations in sensitivity, however, another possibility is that radiologists might have perceptual biases—systematic misperceptions of visual stimuli. Although a great deal of research has investigated radiologist sensitivity, very little has explored the presence of perceptual biases or the individual differences in these.

**Methods:**

Here, we test whether radiologists’ have perceptual biases using controlled artificial and Generative Adversarial Networks-generated realistic medical images. In Experiment 1, observers adjusted the appearance of simulated tumors to match the previously shown targets. In Experiment 2, observers were shown with a mix of real and GAN-generated CT lesion images and they rated the realness of each image.

**Results:**

We show that every tested individual radiologist was characterized by unique and systematic perceptual biases; these perceptual biases cannot be simply explained by attentional differences, and they can be observed in different imaging modalities and task settings, suggesting that idiosyncratic biases in medical image perception may widely exist.

**Discussion:**

Characterizing and understanding these biases could be important for many practical settings such as training, pairing readers, and career selection for radiologists. These results may have consequential implications for many other fields as well, where individual observers are the linchpins for life-altering perceptual decisions.

## Introduction

Medical image perception is fundamentally important for decisions that are made on a daily basis by clinicians in fields ranging from radiology and pathology to internal medicine (Samei and Krupinski, 2018). At a fundamental level, the kinds of decisions that are made depend on the perceptual information that is available to these clinicians (Kundel, 2006; Samei and Krupinski, 2009; Krupinski, 2010). This hinges largely on the clinicians’ basic perceptual abilities as human observers (Kundel, 1989; Quekel et al., 1999; Donald and Barnard, 2012), as well as their specific training and experience (Fletcher et al., 2010; Theodoropoulos et al., 2010; Sha et al., 2020).

It has been known for decades that radiologists have significant individual differences in their diagnostic performance (Elmore et al., 1994; Feldman et al., 1995; Beam et al., 1996; Elmore et al., 1998, 2002; Lazarus et al., 2006; Tan et al., 2006; Elmore et al., 2009; Pickersgill et al., 2019; Sonn et al., 2019). For example, radiologists vary in the accuracy of their mammography reading (e.g., Feldman et al., 1995; Beam et al., 1996; Elmore et al., 2002; Tan et al., 2006). Similar results were found in prostate magnetic resonance imaging screening (e.g., Pickersgill et al., 2019; Sonn et al., 2019). Some studies suggested that these strong individual differences are due to variation in radiologists’ training (e.g., Linver et al., 1992; Berg et al., 2002; Van Tubergen et al., 2003), as well as their experience level (e.g., Herman and Hessel, 1975; Elmore et al., 1998; Manning et al., 2006; Molins et al., 2008; Rosen et al., 2016). Other studies proposed that some differences may be due to the strategies adopted by radiologists (Kundel and La Follette Jr, 1972; Kundel et al., 1978; Krupinski, 1996). For example, radiologists tend to follow two main search strategies. “Drillers” keep fixation on a certain area, and scroll through depth, whereas “Scanners” scan an entire image before moving to the next one (Drew et al., 2013; Mercan et al., 2018).

In recent years, more and more studies have documented and investigated the individual variations in the perceptual performance among groups of untrained observers (e.g., Wilmer et al., 2010; Kanai and Rees, 2011; Wang et al., 2012; Schütz, 2014; Wexler et al., 2015; Grzeczkowski et al., 2017; Wilmer, 2017; Canas-Bajo and Whitney, 2020; Cretenoud et al., 2020; Wang et al., 2020; Cretenoud et al., 2021) and a few studies also investigated the perceptual abilities among clinicians including radiologists (see Waite et al., 2019 for a review; Smoker et al., 1984; Corry, 2011; Birchall, 2015; Langlois et al., 2015; Sunday et al., 2017, 2018). Typical human observers actually have substantial individual differences in their perceptual abilities and biases (for reviews, see Grzeczkowski et al., 2017; Mollon et al., 2017; Wilmer, 2017). These individual differences have been documented from the very lowest level perceptual functions, including localization, motion, and color perception (Schütz, 2014; Wexler et al., 2015; Kosovicheva and Whitney, 2017; Kaneko et al., 2018; Emery et al., 2019; Wang et al., 2020) to higher-level object and face recognition skills (Wilmer et al., 2010; Richler et al., 2019; Canas-Bajo and Whitney, 2020; Cretenoud et al., 2020, 2021). For example, we localize objects nearly every moment of every day, making saccades and other eye movements to the text on this page, reaching for a pen or a coffee cup, or appreciating the position of a pedestrian stepping off a curb into the road. Despite the extensive training in localizing objects, individual observers have strong, stable, and consistent idiosyncratic biases in the locations they report objects to be (Kosovicheva and Whitney, 2017; Wang et al., 2020).

Another example of striking individual differences is face recognition, which varies substantially between observers (Duchaine and Nakayama, 2006; Russell et al., 2009; Wilmer et al., 2010; Russell et al., 2012; Wang et al., 2012; Bobak et al., 2016). For example, so called “super recognizers” can match the identity of random photographs of children to their corresponding adult photographs, whereas those with prosopagnosia often cannot recognize the identity of faces, even themselves or loved ones (Duchaine and Nakayama, 2006; Klein et al., 2008; Russell et al., 2009). These individual differences arise despite extensive training and everyday experiences observers have with faces, and despite the many brain regions and networks devoted to the processing of faces (Kanwisher et al., 1997; Gauthier et al., 2000; Haxby et al., 2001). Holistic face recognition, inversion effects, fractured faces, and other kinds of illusions demonstrate the richness, sophistication, and specialization that we have for recognizing faces (Moscovitch et al., 1997; Farah et al., 1998; Maurer et al., 2002; Rossion, 2013). Still, despite all of that training and exposure, human observers have wildly different face recognition abilities. A great deal of the individual differences in human visual perception might be explained by genetic variations (Wilmer et al., 2010; Zhu et al., 2010; Wang et al., 2018; Zhu et al., 2021), but other individual differences are due to training and experience (Germine et al., 2015; Chua and Gauthier, 2020; Sutherland et al., 2020).

This body of recent perceptual research provides important insights for the idiosyncrasies among radiologists. A first possibility is that there are differences in *perceptual sensitivity*^1^ including visuospatial skills and novel object recognition abilities between clinicians (Smoker et al., 1984; Corry, 2011; Birchall, 2015; Langlois et al., 2015; Sunday et al., 2017, 2018), just like individuals vary in their sensitivity when recognizing faces (Wilmer et al., 2010). This has been the major focus of previous studies investigating individual differences in radiologist perception (see Waite et al., 2019 for a review). These differences in *sensitivity* could be a natural consequence of variability in experience and training (Herman and Hessel, 1975; Linver et al., 1992; Elmore et al., 1998; Berg et al., 2002; Van Tubergen et al., 2003; Manning et al., 2006; Molins et al., 2008; Rosen et al., 2016). Other potential factors include genetic variations, and are not unexpected and could be superseded by training (Bass and Chiles, 1990). A second non-exclusive possibility is that there are differences in *perceptual biases* between different clinicians. For example, clinicians might systematically and consistently misperceive textures, colors, shapes and locations in different ways, as it is known to occur in untrained observers (Schütz, 2014; Wexler et al., 2015; Kosovicheva and Whitney, 2017; Kaneko et al., 2018; Emery et al., 2019; Canas-Bajo and Whitney, 2020; Cretenoud et al., 2020; Wang et al., 2020; Cretenoud et al., 2021).

Whether there are idiosyncratic perceptual biases that clinicians bring to medical image recognition tasks has not been closely studied, but any biases that exist could influence accuracy, diagnostic errors, etc., even if perceptual sensitivity was constant. Conversely, the individual differences in perceptual sensitivity among radiologists (Birchall, 2015; Sunday et al., 2017, 2018) do not predict that there are necessarily systematic idiosyncratic perceptual biases. In fact, there may be no idiosyncratic biases in perception despite the individual differences in accuracy. This is worth reiterating: individual differences in sensitivity need not be the same as individual differences in bias (even if they could be correlated suggested by Wei and Stocker, 2017). Therefore, the question of idiosyncratic biases in clinician perception remains unknown and untested in prior literature.

One reason we believe that investigating perceptual biases (as opposed to sensitivity) was difficult in prior research is that the stimuli used were natural (clinical settings) and therefore not easily or well controlled. Hence, it is almost impossible to measure systematic perceptual biases in radiologists in those studies. In order to measure these idiosyncratic biases in the medical image perception performance of radiologists, we need controlled stimuli and experiments. The goal of this study was to test for idiosyncratic perceptual biases in a group of radiologists with controlled visual stimuli. We also compared the radiologists’ results to a comparable group of naïve participants who were untrained and inexperienced with medical images.

## Experiment 1

Raw data for Experiment 1 were obtained from a previously published experiment on perceptual judgments by radiologists and untrained non-clinical observers (Manassi et al., 2021).

### Methods

#### Participants

Fifteen radiologists (4 female, 11 male, age: 27–72 years) and eleven untrained college students (7 female, 4 male, age: 19–21 years) were tested in the experiment. Radiologists participated on site at RSNA annual meeting and college students were recruited at the University of California, Berkeley. Two radiologists did not finish the study, and their data were excluded. Sample size was determined based on radiologists’ availability at RSNA and was similar to previous studies on the perceptual performance of radiologists and individual differences in visual perceptual biases (Kosovicheva and Whitney, 2017; Manassi et al., 2019; Wang et al., 2020; Manassi et al., 2021). Experiment procedures were approved by and conducted in accordance with the guidelines and regulations of the Institutional Review Board at University of California, Berkeley. Participants all consented to their participation in the experiment.

#### Stimuli and design

**Figure 1:**
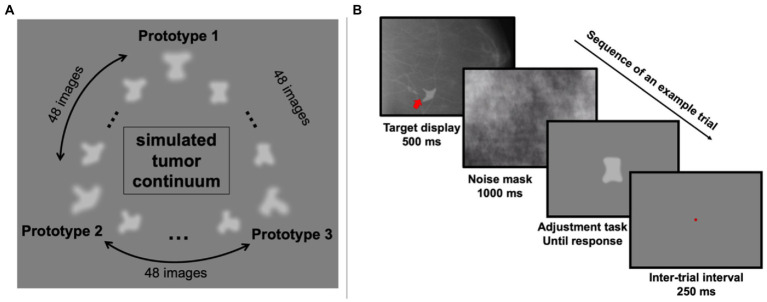
Stimuli and an example trial in Experiment 1. **(A)** The simulated tumor continuum was generated from three tumor-shape prototypes. Between each pair of prototype images, 48 morph images were generated, resulting in a total of 147 images. **(B)** On each trial, observers saw a simulated tumor target (indicated by the red arrow; note that the red arrow was never shown in actual experiment) superimposed on a real radiograph for 500 ms, which was followed by a noise mask covering the full screen for 1,000 ms. Then a randomly chosen simulated tumor was shown at the center of the screen and observers pressed the left or right arrow key to adjust it and match it with the target. They confirmed their response by hitting the space bar and after a 250-ms inter-trial-interval, the next trial began.

Three random objects were created to simulate tumor prototypes. Between each pair of prototypes, 48 morph images were generated using FantaMorph (Abrosoft Co.). This resulted in a continuum of 147 simulated tumors in total (Figure 1A). In addition to the simulated tumors, 100 real mammogram images taken from The Digital Database for Screening Mammography were used in this study as background textures (Bowyer et al., 1996).

On each trial, one of the 147 simulated tumors was randomly chosen and presented on top of a randomly chosen real mammogram background image (see Figure 1B for an example trial). The simulated tumor was shown at a random angular location relative to central fixation (0.35 degrees of visual angles) in the peripheral visual field with an eccentricity of 4.4 degrees of visual angle. After 500 ms, a noise mask covered the whole screen for 1,000 ms to reduce retinal afterimages. Next, one random simulated tumor image was shown at the center of the screen, and participants (trained radiologists and untrained observers) were instructed to adjust the current image to match the previously shown simulated tumor. This adjustment was performed by pressing the left and right arrow keys to move along the simulated tumor continuum. Participants were allowed to take as much time as needed to complete this task. Once they decided on the chosen image, they confirmed their response by pressing the space bar. A brief 250 ms pause followed their response, and then the next trial began. Each participant completed 255 trials in total.

### Data analysis

For each participant, we estimated their perceptual biases with their response errors on each trial by calculating the shortest distance in morph unit on the simulated tumor continuum between the target and their response.

In order to directly compare the discriminability of the simulated tumors between radiologists and untrained observers, we calculated the just-noticeable-difference (JND) by fitting a Gaussian function on the response error frequency on individual observers, and calculated half of the distance between the 25th and 75th percentile of the cumulative Gaussian distribution that was transformed from the best-fitted Gaussian function.

**Figure 2:**
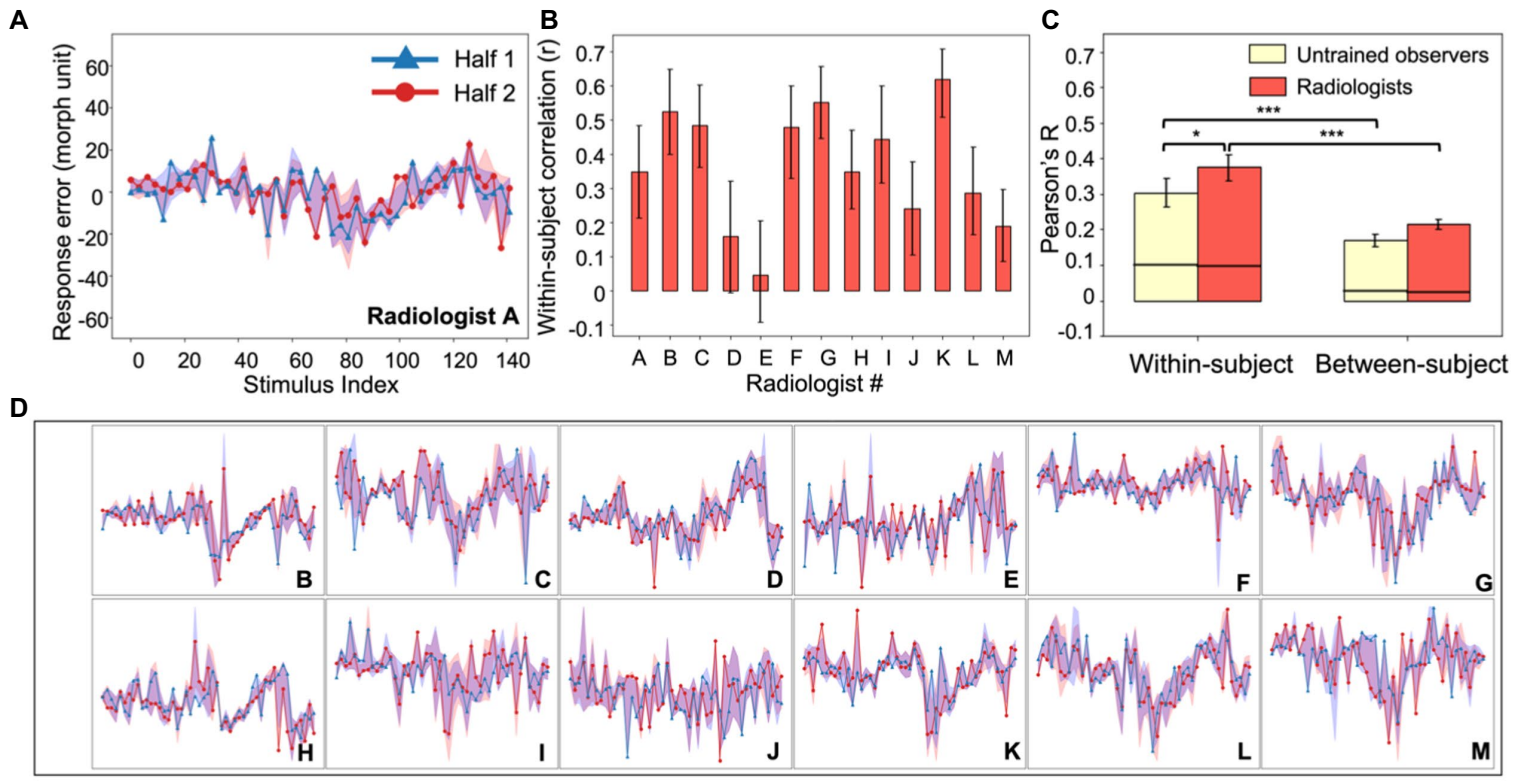
Experiment 1, individual differences in radiologists’ perception. **(A)** An example radiologist’s error plot. The abscissa shows the stimulus index (from Figure 1A) and the ordinate is the observer’s continuous report error. Because of the limited number of trials, data were down-sampled by binning every three neighboring simulated tumors on the continuum into one; this resulted in 49 instead of 147 data points (see Experiment 1, Data Analysis). For the purposes of visualization, we randomly split the data into two halves (red and blue curves) and plotted them separately (in the analysis this procedure was repeated, see Methods). The shaded area around the two halves represents the 95% bootstrapped confidence interval for each half. **(B)** The average bootstrapped split-half within-subject correlation for each radiologist. Error bars represent the 95% bootstrapped CIs for each individual radiologist. **(C)** Within-subject consistency and between-subject consistency were averaged within each group of observers (radiologists in red; untrained observers in yellow). For both groups, the within-subject correlations were significantly higher than between-subject correlations, and radiologists were significantly more consistent within themselves compared to untrained observers. Error bars represent the 95% bootstrapped CIs and the horizontal black lines represent the 97.5% upper bounds of the permuted null distributions. *p < 0.05, ***p < 0.001. **(D)** Individual radiologist’s error plots for Radiologists B-M. Strong idiosyncrasies are clear between different radiologists while at the same time there are noticeable consistencies within each radiologist, indicating stable response biases. The abscissa and ordinates for error plots in **(D)** are exactly the same as those in **(A)** so for visual simplicity, they are not labeled.

**Figure 3:**
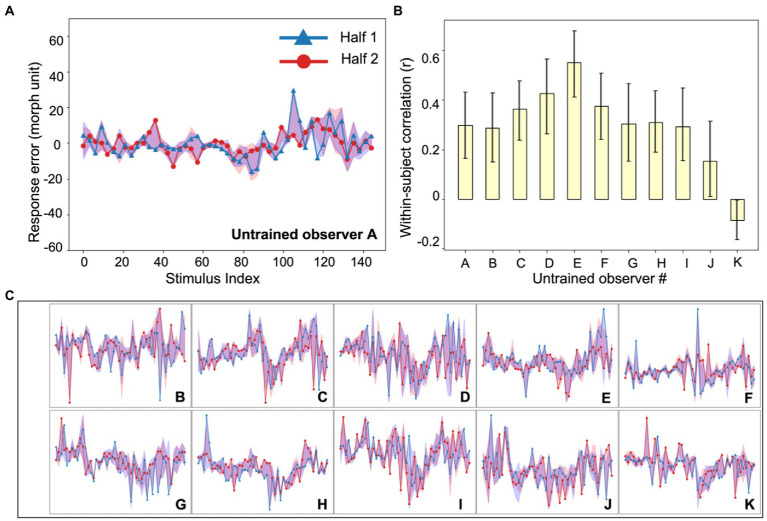
Experiment 1, individual differences in untrained observer perception of simulated tumors. **(A)** An example untrained observer’s error plot (as in Figure 2). The shaded ribbons around the two halves (red and blue data) represent the 95% bootstrapped confidence intervals for each half. **(B)** The bootstrapped within-subject correlation for each untrained observer (c.f., the group average in Figure 2C). One observer (Observer K) showed a moderately negative within-subject correlation, but this could be due to chance. Error bars represent the 95% bootstrapped CIs. **(C)** Individual subject error plots for untrained observers B-K. The individual differences previously found among radiologists (Figure 2) were replicated with the untrained observers. Ordinate and abscissa for each error plot in **(C)** are identical to those in panel **(A)**.

Within-subject consistency in the response errors was calculated with a split-half correlation for each observer. To compensate for the lack of trials for each image, we first binned every three simulated tumors into one, so that the number of unique simulated tumors was reduced to 49, but every binned simulated tumor had on average 5 trials of response errors. We then used a nonparametric bootstrap method to estimate split-half correlations (Efron and Tibshirani, 1994). On each iteration, for each observer and each binned simulated tumor, we randomly split the responses into two halves and calculated the mean response errors for each half (see Figures 2A,D for the two randomly-split halves from all radiologists and Figures 3A,C for all untrained observers). Next, the two halves were correlated and then the Pearson’s r value was transformed into a Fisher z value (see Figures 2B, 3B for the individual within-subject correlations for each radiologist and each untrained observer). We then averaged the z values from radiologists and untrained observers separately and the averaged Fisher z values from two groups were transformed back to Pearson’s r values (*Fisher* transformations were applied for all analyses when calculating the average of correlation values). We repeated this procedure 1,000 times so that we could estimate the mean within-subject correlations and 95% bootstrapped confidence intervals (CI) for radiologists and untrained observers separately (Figure 2C, left panel).

Between-subject consistency was calculated similarly. After splitting every observer’s data into two random halves (i.e., by randomly selecting 50% of the data on each iteration), we correlated one half from one observer with one half from another observer. All pairwise correlations were averaged to estimate the between-subject consistency. By repeating the procedure 1,000 times, we obtained the mean between-subject correlations and 95% bootstrapped CIs separately for radiologists and untrained observers (Figure 2C, right panel).

Next, we estimated the expected chance-level within and between-subject correlations by calculating permuted null distributions. On each iteration, and for each observer, we again split the response errors for each binned simulated tumor into two halves as we did in the bootstrap procedure. We then systematically shifted one half by some random units (for example, simulated tumors 1, 2, 3 might be labeled as simulated tumors 7, 8, 9), and the shifted half was correlated with another unchanged half. For within-subject correlations, the unchanged half came from the same observer. For the between-subject correlations, the unchanged half came from a different observer. The resulting correlations from individual participants (within-subject) or different pairs of participants (between-subject) were averaged together to get the permuted within-subject or between-subject correlations. This permutation method allowed us to estimate the null correlations by correlating the response errors of different stimuli with each other while at the same time preserving the relationship between similar stimuli (*Monte Carlo* Permutation Test, MCPT, Dwass, 1957; Edgington and Onghena, 2007; Manly, 2018). This permutation procedure was repeated 10,000 times to estimate permuted null distributions for within-subject and between-subject consistency. We did this separately for radiologists and untrained observers. The mean empirical bootstrapped correlations were then compared to their corresponding permuted null distributions to estimate the statistical significance of the mean bootstrapped within and between-subject correlations.

Internal consistency of the stimuli used in the experiment was calculated using Cronbach’s alpha (Cronbach, 1951). We first binned the simulated tumors into three categories (i.e., the three prototypes). Each image was labeled as the closest simulated tumor prototype based on its distance on the simulated tumor continuum. Participants’ responses (i.e., selected images) were also transformed into three categorical responses as above. We estimated Cronbach’s alpha separately for radiologists and untrained observers.

## Results

Our goal was to measure individual differences in radiologist perception and compare these to the individual differences found in a sample of untrained observers. In Experiment 1, we used artificial radiographs: images with controlled shapes that were presented briefly in noise (Figure 1A). The background noise was taken from authentic radiographs (Bowyer et al., 1996), and was therefore realistic. The simulated tumors, on the other hand, were intentionally artificial because we aimed at having highly controlled stimuli with solid ground truth information; this allowed us to precisely measure perceptual biases in judgment. On each trial, the clinician saw a brief image of a radiograph with a simulated tumor. The clinician was asked to find the simulated tumor in the noise image, and then to match that simulated tumor with a test stimulus in a continuous report paradigm (Figure 1B). The advantage of this paradigm over categorization or forced-choice tasks is that it gives trial-wise errors and allows us to measure a complete error distribution with high-resolution information. The goal here was not to recreate a diagnostic imaging task, but to measure perceptual biases for visual stimuli that used noise backgrounds similar to those found in typical radiographs.

The results showed that the practicing radiologists are able to match the artificial tumor with a corresponding shape very accurately: mean JND was 10.5 morph unit with standard deviation 2.0 morph unit). This confirms that they were able to detect and recognize the simulated tumors. Our goal was to look for individual differences that may have been stable and consistent within a particular observer—whether there are idiosyncrasies in clinician perception. To measure this, we calculated the consistency in the observer judgments of the simulated tumors. Each simulated tumor was different, and we measured systematic errors in judgments for each specific image. Insofar as there are differences in clinician perception, they might report deviations or biases and (mis)report a simulated tumor consistently.

Figure 2A shows an example of an individual radiologist’s biases as a function of stimulus number and all remaining radiologists are shown in Figure 2D. We calculated the split half correlation within each observer (Figure 2B) across all of the stimuli and found that there was a significant within-participant correlation (Figure 2C, left panel, red bar; mean Pearson’s *r* = 0.37, *p* < 0.001, permutation test). Hence, each observer had idiosyncratic biases in their perceptual reports, and those were consistent within each observer. We also calculated the between-observer correlation, using the same approach. This is the correlation between different clinicians, or how similar their residual errors were to each other; it is a measure of how much agreement there is between observers. We found that there was significantly more correlation within a given clinician than between clinicians (Figure 2C, right panel, red bar; mean *r* = 0.22, bootstrap test, *p* < 0.001). This cannot be attributed to noise. Simply adding noise reduces the correlation both within and between observers; adding noise cannot increase the within observer correlation. The results suggest that individual clinicians have consistent biases in their perceptual reports. The source of these biases is unclear, but they are observer-specific.

To compare this sample of clinicians with an untrained group, we collected data on the same experiment with another group of naive untrained non-clinical observers (Figure 3). The observers performed the exact same continuous report matching task. Untrained observers also perceived the simulated tumors accurately (mean JND: 10.0 morph unit, standard deviation: 1.9 morph unit; see Experiment 1, Data Analysis for the estimation of JNDs) and their discriminability did not differ from that of radiologists (*t* = 0.64, *p* = 0.53). We also looked into the within-subject and between-subject consistency among untrained observers and found qualitatively similar results (Figure 2C). First, there were significant individual differences in the untrained observers (Figure 2C, left panel, yellow bar; mean *r* = 0.30, *p* < 0.001, permutation test; individual observer within-subject correlations in Figure 3B). The between-subject correlation was significantly lower (Figure 2C, right panel, yellow bar; mean *r* = 0.17, *p* < 0.001, bootstrap test). This echoes the group of radiologists: there are individual differences in simulated tumor recognition, even in untrained observers.

There are, however, several differences between the radiologists and untrained observers that are worth noting. First, the within-observer correlation was higher for the radiologist group than for the untrained observers (*p* < 0.05, bootstrap test). Clinicians are more consistent in their observer-specific biases than untrained observers. Given that clinicians and untrained observers do not differ significantly in their perceptual sensitivity measured by JNDs, this result echoes our hypothesis that idiosyncratic perceptual biases could be observed even without differences in overall perceptual sensitivity. Second, the between-subject correlation was not 0 in either group (*ps* < 0.001, permutation test). There are therefore some consistencies between observers in how these stimuli are judged. The individual differences, however, significantly outweighed the commonality, since the within-subject correlations of both groups were significantly higher than the between-subject correlations (*ps* < 0.001). Together, the results in Experiment 1 showed that radiologists and untrained observers both demonstrated strong individual differences in their perceptual biases towards different simulated tumors in a shape matching task, and radiologists tend to have higher consistency in their own biases.

However, several questions were still left unanswered. First, in Experiment 1, although we used real mammograms as backgrounds, the simulated “tumors” were clearly artificial and different from real tumor shapes (Figure 1A). It is unclear whether radiologists would show any idiosyncratic perceptual biases on real or very-close-to-real radiographs. Second, it remains unknown whether these perceptual biases can be observed in perceptual tasks other than continuous report shape matching. Third, we wondered whether similar individual differences would still exist for medical images other than mammograms. Therefore, to further explore these questions, we conducted a second experiment.

## Experiment 2

Raw data for Experiment 2 were obtained from a published study (Ren et al., 2022).

### Methods

#### Participants

Seven trained radiologists (3 female, 4 male, age: 28–40 years) and five untrained observers (3 female, 2 male, age: 23–25 years) were recruited in the experiment. Sample size was determined based on previous studies on the perceptual performance of radiologists and individual differences in visual perceptual biases (Kosovicheva and Whitney, 2017; Manassi et al., 2019; Wang et al., 2020; Manassi et al., 2021). Experiment procedures were approved by and conducted in accordance with the guidelines and regulations of the Institutional Review Board at University of California, Berkeley. Participants all consented to their participation in the experiment.

#### Stimuli and design

**Figure 4:**
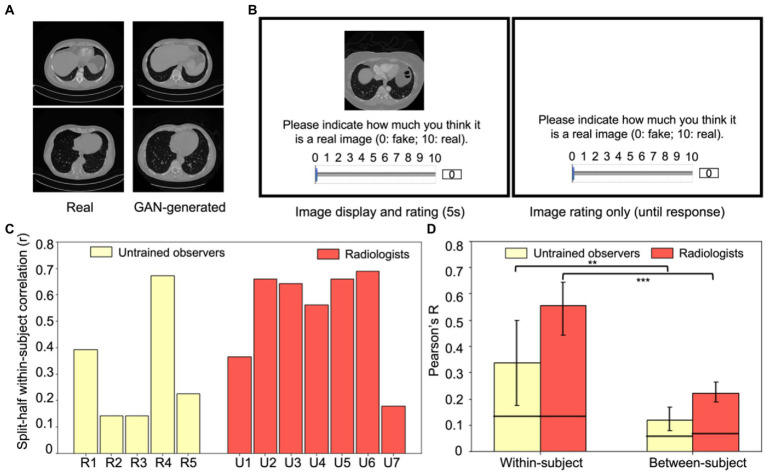
Experiment 2 example stimuli and results. **(A)** Examples of real (left column) and GAN-generated (right column) CT lesion images used in the experiment. Visually and qualitatively the images are similar, and a previous study showed that these GAN-generated images were visually metameric: they were indistinguishably realistic to both radiologists and untrained observers (Ren et al., 2022). **(B)** An example trial in Experiment 2. On each trial, either a real CT lesion image or a GAN-generated image was shown on the screen for at most 5 s. Participants could rate the realness of the image from 0 to 10 (0: fake, 10: real) anytime during the image presentation or after the disappearance of the image (self-paced). The next trial began after the rating was made. **(C)** Within-subject consistency for each radiologist and untrained observer, calculated from the split-half correlation of each observer’s response errors of the same images. **(D)** Within-subject correlations significantly exceeded between-subject correlations for both groups (*p* < 0.01 for untrained observers and *p* < 0.001 for radiologists), which replicated the individual differences found in Experiment 1 and extended the results to a new task, a new type of medical image, and with more realistic stimuli. Error bars represent the 95% bootstrapped CIs and the horizontal lines represent the 97.5% upper bounds of the permuted null distribution. **p < 0.01, ***p < 0.001.

Fifty CT lesion images were randomly sampled from the DeepLesion Dataset (Yan et al., 2018), and fifty simulated lesion images were generated through the Generative Adversarial Networks (GAN) trained with 20,000 real CT lesion images from the DeepLesion Dataset (Ren et al., 2022). This resulted in a total of 100 images (see Figure 4A for examples). According to Yan et al. (2018), there were multiple types of lesions in the DeepLesion Dataset, including lung nodules, liver tumors, enlarged lymph nodes, and so on, and images included both chest and abdomen CT images.

Both radiologists and untrained observers were recruited to perform an image rating task (Figure 4B). On each trial, one of the 100 images was pseudo-randomly chosen to present to the observers and it remained on the screen for at most five seconds. Observers were instructed to rate the realness of the image on a continuous scale ranging from 0 to 10 (0: fake, 10: real). Participants could respond at any point during image presentation, or they could take as much time as necessary after the image offset. The next trial started immediately after their response. Each image was shown exactly once in these 100 trials. Both radiologists and untrained observers were informed that the stimuli shown in the experiment were composed of 50% real images and 50% GAN-generated images.

To estimate test–retest reliability, 20 real images and 20 GAN-simulated images were randomly chosen from the aforementioned 100 images. These 40 images were randomly inserted in the previous 100 image list and were presented in the same manner. Thus, there were in total 140 trials for each participant.

### Data analysis

Due to a technical problem during image display, one of the 40 repeated images failed to show up for some participants, so only the ratings for 39 out of the 40 repeated images were used (39 initial ratings and 39 retest ratings) in all following analyses.

We recognized that the raw ratings could be influenced by participants’ extreme response tendencies. For example, some might tend to give higher ratings across all images while some may rate lower. Throughout the manuscript we refer to these types of response tendencies as “response propensities,” to avoid confusion with other terms like response bias, that can mean different things in different circumstances. To reduce the effect of response propensities, for each participant, we first normalized their raw ratings by rescaling them to range from 0 to 10 using the equation below ( X is the raw ratings from one participant, $X_{\min}$ is the minimum of this participant’s raw ratings and $X_{\max}$ is the maximum).


$$X_{new}=\frac{10*\left(X-X_{\min}\right)}{\left(X_{\max}-X_{\min}\right)}$$

After normalization, for each participant, we again used response errors as a proxy for perceptual biases. We estimated their response errors by calculating the absolute difference between the normalized ratings and the corresponding ground truth of each image (0 for GAN-generated images and 10 for real CT images). Then, similar to Experiment 1 (see *Data Analysis*), we estimated the within-subject and between-subject consistency of their response errors. Within-subject consistency was estimated by the average test–retest reliability among participants (i.e., correlating the response errors from the 39 initial trials and the 39 retest trials). Figure 4C shows the individual split-half within-subject correlations for each radiologist and each untrained observer. Between-subject consistency was estimated as the average pairwise correlations among participants. This was calculated separately for radiologists and untrained observers.

Bootstrap distributions of the within and between-subject correlations were estimated to test whether the average correlations were simply driven by extreme observer(s). For within-subject correlations, on each iteration, we randomly sampled seven radiologists and five untrained observers with replacement, calculated each observer’s within-subject correlation and then averaged the correlations through Fisher transformation (Figure 4D, left panel). For between-subject correlations, on each iteration, we sampled the same number of pairs of subjects from all possible pairs of subjects with replacement, calculated all pairwise correlations for the sample and the between-subject correlation was estimated as the mean of all pairwise correlations (Figure 4D, right panel). These procedures were repeated 1,000 times to estimate the 95% within-subject and between-subject bootstrapped CIs for radiologists and untrained observers separately.

To examine the expected correlations by chance, permuted null distributions were also calculated in Experiment 2 by shuffling the image labels for the initial trials and the retest trials, so that the response error for one image might then be treated as the response error for another. Null distributions were separately calculated for radiologists and untrained observers. On each iteration, the shuffled response errors from initial trials and retest trials from the same observer were correlated to obtain a null estimate of within-subject consistency. This was done for every observer (every radiologist and every untrained observer), and the set of pairwise correlations were averaged across the group of observers to create one null sample. This procedure was repeated 10,000 times to create a null within-subject distribution. To create between-subject null distributions, we calculated all pairwise correlations between the shuffled initial response errors from one observer and the shuffled retest response errors from another observer. This was repeated 10,000 times to generate a between-subject null distribution. Permuted null distributions were calculated separately for radiologists and untrained observers, and, from these, 95% permuted confidence intervals (CIs) were estimated.

## Results

In Experiment 2, we tested whether idiosyncratic perceptual biases can be observed with images from a different modality (CT images), a very different perceptual task and highly realistic GAN-generated images. Generative Adversarial Networks (GAN) were trained by Ren et al. (2022) with real CT lesion images taken from the DeepLesion Dataset (Yan et al., 2018), and then the GAN model was used to generate artificial lesion images. Observers were recruited to perform an image discrimination task including 50 real lesion images and 50 GAN-generated images (see Figure 4A for example stimuli), in which both radiologists and untrained observers rated how realistic each image appeared. Among these 100 images, 20 real images and 20 artificial images were repeated twice so that we could estimate the within-subject consistency. Figure 4B shows an example trial.

In general, the GAN-generated images were indistinguishable from real lesion images for both untrained observers and radiologists (mean *d’* values: 0.18 and 0.27 respectively) and there was no significant difference between different groups of participants (*t* = 0.4, *p* > 0.5). This suggested that the artificial GAN-generated images were highly realistic and even experts with training could not distinguish them effectively (Ren et al., 2022). This general lack of sensitivity, however, does not preclude individual biases in the discrimination of the CT lesion images.

The goal of the following analysis was to measure whether there are systematic and idiosyncratic stimulus-specific biases in the perception of CT lesions by radiologists and untrained observers. As in the first experiment, we measured within and between subject consistency of the perceptual judgments. Since raw ratings may be subject to observers’ response propensities, we normalized the ratings for each observer and then calculated response errors to get a more accurate estimate of their perceptual biases based on the normalized ratings (see Experiment 2, Data Analysis). Figure 4C shows each observer’s within-subject consistency and the mean within-subject and between-subject consistency is shown in Figure 4D. We again found a significantly higher within-subject consistency (Figure 4D, left panel) in both radiologists (Pearson’s *r* = 0.56) and untrained observers (Pearson’s *r* = 0.34) compared to their corresponding between-subject consistencies (Figure 4D, right panel; *r* = 0.22 and *r* = 0.12, bootstrap test, *p* < 0.001 and *p* < 0.01, for radiologists and untrained observers respectively). This replicated the findings in Experiment 1, indicating that each radiologist and each untrained observer have their own unique biases in the perception of medical images that cannot be explained by shared biases among observers. We again found that between-subject correlations were significantly higher than the permuted null correlations for both groups of observers (permutation test, *p* < 0.001 and *p* < 0.01 for radiologists and untrained observers respectively), suggesting that observers do share some of their biases. This could be due to some textures or features of the images that commonly influenced the observers’ discrimination of the real or fake CT lesion images.

Taken together, in Experiment 1 we found idiosyncratic biases in radiologists when they made perceptual judgments about artificial simulated tumor shapes, and their biases were stronger compared to untrained observers. Experiment 2 further extended these results and demonstrated strong individual variations in radiologists’ biases in the perceived realness of GAN-generated and real CT lesion images, suggesting that idiosyncratic perceptual biases among radiologists are not tied to a specific type of medical images or tasks, but rather they can be generally observed among different modalities of medical images and different tasks. These individual observer specific biases are found even without significant difference between observers’ perceptual sensitivity measured by *d’*.

## Discussion

We found significant individual differences in radiologists’ perceptual biases. Experiment 1 showed that each radiologist demonstrates unique perceptual biases towards simulated tumors in a shape matching task, and their own internal biases were even more consistent than untrained observers (Figure 2C). Experiment 2 replicated and extended the results by again showing individual differences in radiologists when they perceived GAN-generated and real CT lesion images in an image discrimination task (Figure 4D). These individual differences were not simply induced by task or stimuli since they were found across different radiologists, different modalities of medical images, and different tasks. Thus, we propose that the individual differences in radiologist perception may arise at least in part because of distortions in perceptual judgments at the level of specific clinicians. These kinds of individual differences in basic perceptual bias could, in turn, potentially influence the performance of radiologists in diagnostic practice.

What are the potential mechanisms underlying these idiosyncratic perceptual biases among radiologists? One possibility is that different radiologists may have different perceptual templates or perceptual representations of the tumor or of medical images in different modalities, analogous to studies showing that human observers have different perceptual templates of faces (e.g., Dotsch and Todorov, 2012; Moon et al., 2020). Differences in these templates could be associated with different biases, and could arise as a natural consequence of their intensive training and years of experience (Imhoff et al., 2011; Jack et al., 2012; Soto, 2019). Previous research has supported that attention towards perceptual stimuli can be guided differently according to the perceptual templates or mental representations (e.g., Griffin and Nobre, 2003; Olivers et al., 2011), so the variations in observers’ mental representations could potentially direct their attention to different parts of the simulated tumors or the radiograph background and thus lead to idiosyncratic perceptual biases. It remains unknown if this is the case, but it is worth pursuing in future research.

Another possible explanation is natural statistics. Radiologists may not have literal “templates,” but may have some priors or learned distributions of the statistics in medical images, similar to how human observers represent the statistics of scenes (Torralba and Oliva, 2003; Stansbury et al., 2013). These priors may include low-level information such as luminance and contrast, but may also contain higher-level, multi-dimensional representations of textures. These kinds of image statistics could underlie perception of gist in medical images (Evans et al., 2019). Sensitivity to this information can be shaped specifically by the natural statistics of medical images that clinicians are exposed to during their medical training and diagnostic practice, and it can also come from other non-specific visual images or perceptual experiences in their everyday life. This could explain why the GAN-generated medical images may have confused the experts in Experiment 2, since the image statistics would have been learned and captured by the GAN. Our current study cannot pinpoint the underlying mechanism responsible for idiosyncratic perceptual biases, but image statistics or templates (or both) could be involved. It would be valuable to explore this in future research.

There are several concerns raised by our results that we address here. First, it might be argued that stronger idiosyncratic biases in the radiologists in Experiment 1 could simply result from the radiologist group being more attentive to the task or lapsing less frequently. In principle, that may explain the higher within-subject correlation as well as the higher between-subject correlation in Figure 2C. Although this is possible, this does not seem likely, as the overall discriminative ability was fairly similar between the two groups and the just noticeable difference was comparable and not significantly different for both the clinician group and the naïve untrained group (see Experiment 1 results). Hence, the stronger within-subject correlations—stronger individual differences within the clinicians—did not simply arise because of attentiveness. On the other hand, the stronger individual differences in the clinician group could arise, in part, from the unique and lengthy training that the clinicians receive, or the practice that they have had in related types of perceptual tasks.

One limitation is that the task we used in the Experiment 1 was not realistic and arguably was not representative or typical of a radiologist’s task because radiologists mostly perform detection or categorization tasks in their everyday routine, while our task was a continuous report adjustment method. However, the adjustment task can be more advantageous than detection or categorization tasks since it can measure the subjective perceptual representations and criterion of the observers (Pelli and Farell, 1995), it provides very fine-grained information about errors, and it provides critical behavioral insights for understanding the perceptual biases in medical image perception. Moreover, there is no evidence that we know of that continuous report psychophysical measures systematically misrepresent recognition processes (e.g., Prinzmetal et al., 1998; see Stevens, 1958; Gescheider, 2013 for reviews). Nevertheless, future studies could extend our results by testing radiologists with our controlled realistic stimuli in a task that is more similar to those in clinical practice.

Another related concern is that the task in Experiment 1 may be unrealistic because it required a variety of perceptual and memory related skills. Observers (naïve observers or skilled radiologists) were asked to detect and recognize a simulated tumor. During this task, they had to hold information in visual short-term memory and subsequently match a stimulus to what was previously seen. This is indeed broader in scope than a traditional forced-choice paradigm. Nevertheless, the detection, recognition, and visual short term memory processes involved in our task are the kinds of abilities that are used by clinicians on a daily basis. Multitasking is not uncommon for radiologists in realistic settings; they often have multiple screens and multiple radiographs; they gaze between different regions of the visual field and integrate information separately from multiple radiographs and files; radiologists often need to hold in short term memory information about the patient, diagnostic history, etiology, referring physician, etc.; and, they may be interrupted mid-diagnosis by the phone, noise, and other realistic factors (see Kansagra et al., 2016, for a review). Moreover, visual short term memory processes work even at the shortest time scales (e.g., even across saccades; Irwin, 1991). In other words, the complexity of the radiologist’s task goes well beyond a simple instantaneous forced-choice.

Our experiment does not capture the full complexity of the radiologist’s family of tasks, but the basic processes it taps are highly relevant to those used by radiologists. The results reinforce this: radiologists had higher within-subject consistency than the untrained observers. This suggests that individual radiologists have more consistent and systematic biases in this simulated tumor matching task compared to untrained observers, indicating that their expertise or experience is in fact reflected in this task. Although radiologists and untrained observers had similar sensitivity, as measured by JNDs, this is not surprising since previous studies have found that untrained, naïve observers can perform significantly better than chance in the Vanderbilt Chest Radiograph Test (Sunday et al., 2017), and other studies found that MDs are not always more sensitive than untrained participants in medical image perception tasks, and sometimes they even have lower sensitivity compared to less experienced observers (e.g., Wolfe, 2022). The similar sensitivity in MDs and untrained observers in our experiment could be due to a ceiling effect in our data, but the fact that the consistency in reports is higher for MDs suggests that they do, in a sense, perform the task better than untrained observers.

One way to address this question about ecological validity is to test whether our results extend to other tasks, especially involving real medical images and stepping beyond the artificial radiographs. Therefore, we analyzed data from a second experiment that used realistic CT lesion images. Although this is a different area of medical image perception, we hypothesized that idiosyncratic perceptual biases can be observed across domains and should not be limited to any particular modality, stimulus, or task. In the second experiment, we used real CT lesion images combined with artificial but realistic lesion stimuli created by Ren et al. (2022). These stimuli (see Figure 4A for example) are different than artificial stimuli in Experiment 1 (Figure 1A) because they were highly realistic, even metameric (completely confusable) with real lesions (Ren et al., 2022). Using those realistic images, we found that both radiologists and untrained observers showed clear individual differences in their perception of the real or fake CT lesion images, which extended our previous findings from a matching task to a real/fake rating task and from artificial shapes to highly realistic CT images. The results from Experiment 2 further supported our hypothesis that observer-specific perceptual biases are not domain modality or task-specific. Rather, they are likely a ubiquitous effect in realistic medical imaging tasks with implications across domains. Therefore, though our tasks might not be the most realistic or cannot be directly linked with diagnostic performance of radiologists, these compelling results clearly demonstrate that even well-trained radiologists can have idiosyncratic and stimulus-specific perceptual biases with medical images under different task settings.

One might still be concerned about the internal consistency for these idiosyncratic biases. Using the split-half Pearson’s correlation, we found that radiologists had an internal reliability of 0.37 (Experiment 1) and 0.42 (Experiment 2). While this may seem somewhat low, it is significantly higher compared to what was expected by chance (i.e., the permuted null distributions) and it may appear low only because our stimuli were numerous and very finely spaced. In order to compare our results with previous published studies, in Experiment 1, we dummy-coded the data into binned categories (like a three-alternative-forced-choice, 3AFC, classification task, see Experiment 1, Data Analysis for details) and the Cronbach alpha rises substantially (alpha = 0.85 for radiologists), and in Experiment 2, the Cronbach alpha for radiologists was 0.95, which are indeed comparable to that reported in a previous study on individual difference in a radiograph-related task (Sunday et al., 2017). This is unsurprising because noise at the individual stimulus level is averaged out and what remains is a less noisy estimate of the more substantial individual differences.

The between-observer consistency is typically the focus of most medical image perception research (see Donovan et al., 2017 for a review; Elmore et al., 1994; Feldman et al., 1995; Beam et al., 1996; Elmore et al., 2002; Lazarus et al., 2006; Tan et al., 2006; Elmore et al., 2009; Donovan and Litchfield, 2013; Sunday et al., 2017, 2018; Pickersgill et al., 2019; Sonn et al., 2019). Recently, a study by Sunday et al. (2017) explored the internal within-observer consistency in medical image perception. Our results complement this from a different perspective, showing that it is equally or even more important to measure individual differences in each radiologically-relevant task by measuring both the within-subject and between-subject consistency in radiologists. Our results also go a step further to compare the individual differences in radiologists with untrained non-clinical observers, and they provide evidence for a stronger idiosyncrasy in radiologists’ perception of artificial and realistic medical images across domains, which was not clear in previous studies on individual differences in radiologists. The stronger within-subject consistency compared to between-subject consistency also provides a direct insight about the relative importance of the individual perceptual variations and shared biases among observers: individual differences are substantial, and can even swamp the between-subject similarities.

The scarcity of the expert radiologist pool undoubtedly limited the number of available observers we were able to test. Although this is a limit in group-wide analyses, we analyzed every individual observer and measured trial-wise effects within each observer. In fact, even when sample size was limited, past research has been able to demonstrate strong and consistent idiosyncratic visual perceptual biases towards object location, size, motion and face perception with the help of psychophysics (Afraz et al., 2010; Schütz, 2014; Wang et al., 2020). Our results aligned with these previous findings and provided new insights of the prevalent idiosyncratic perceptual biases that can be found with medical images and among well-trained radiologist experts.

There are several implications of the findings reported here. First, clinicians vary in their perceptual abilities. Although this is not at all surprising, the stimulus-specific way in which clinicians vary in the perceptual biases is novel. Second, we found that individual differences are not washed out by training. To address this, we performed a Fisher’s combined probability test (Fisher, 1925; Fisher, 1948; Rosenthal, 1978), which combines the statistical results from both Experiment 1 and Experiment 2 in a type of “mini meta-analysis” (Goh et al., 2016). We found that, across the experiments, there is a significantly higher within-subject consistency for radiologists compared to untrained observers ( $\chi_{2}^{2}$ = 12.9, *p* < 0.005). That is to say, counterintuitively, some biases may get *stronger* with training, leading to more stable individual differences within radiologists compared to untrained observers. Combined with the fact that radiologists and untrained observers were not significantly different in terms of perceptual sensitivities (measured by JNDs in Experiment 1 and *d’* in Experiment 2), this result again echoes our hypothesis that variations in perceptual biases could exist even without overall differences in perceptual sensitivity. Third, our results show that even untrained observers bring with them individual biases and idiosyncrasies in their perceptual judgments. Fourth, idiosyncratic distortions were found across two different domains, two different modalities, and two different imaging techniques (see Experiment 1 vs. Experiment 2).

More importantly, the fact that there are individual differences between observers could have critical implications for diagnostic medical imaging. For example, in some countries, it is common practice to have multiple readers rate or diagnose radiographs (Australia, 2002; Yankaskas et al., 2004; Amendoeira et al., 2013). Given that much of the variance in observer judgments is attributable to the individual observer themselves, it may directly influence the employment or selection of the pairs of observers: two observers that have similar individual differences may perform more poorly (since the biases could potentially exaggerate after combining) than two readers who have more independent individual differences. Individual differences that are more independent will tend to cancel out and thus lead to more accurate medical image perception (Van Such et al., 2017; Corbett and Munneke, 2018; Taylor-Phillips and Stinton, 2019). Thus, our results may suggest the importance of measuring perceptual biases in radiologists before grouping them into pairs. We believe this could be a valuable strategy in paired reading, as it goes well beyond simply relying on radiologists’ diagnostic accuracy (e.g., Brennan et al., 2019).

Another important implication of our results is that different clinician observers may show different native ability in particular specialties or even different imaging modalities. Returning briefly to the face recognition literature, the individual differences in face recognition arise because of many factors including age and experience, but also genetic differences (Wilmer et al., 2010; Shakeshaft and Plomin, 2015). Some observers are simply genetically predisposed to be more sensitive to faces. The same may be true in medical image perception. Although we do not know what proportion of the individual differences are accounted for by genetic factors, this will be an important future area of research. Whether or not there is a substantial genetic contribution, the individual differences in clinician perception can also be measured, selected, and trained. Some previous work has already started to address this with mostly focusing on perceptual sensitivity (Corry, 2011; Birchall, 2015; Langlois et al., 2015; Sunday et al., 2017, 2018; see Waite et al., 2019 for a review). The individual differences reported here also echo those found in other domains of perception research (e.g., Wilmer et al., 2010; Wang et al., 2012; Schütz, 2014; Wexler et al., 2015; Grzeczkowski et al., 2017; Canas-Bajo and Whitney, 2020; Wang et al., 2020), and raise the possibility that idiosyncratic distortions in clinician perception may be widespread and extend across different domains.

## Conclusion

Our findings provide a new insight about the individual differences that exist in the perceptual judgments of professional radiologists: apart from perceptual sensitivity, which has been proposed and investigated extensively in the past, there may actually be idiosyncratic and systematic biases in their perceptual judgments. Understanding these idiosyncratic perceptual biases could be critically important for a variety of reasons, including training, career selection, bias compensation, and employing paired readers in the field of medical imaging. At an even broader level, it is worth noting that individual differences in observer perception could have important consequences in many fields beyond medicine. For example, in TSA screeners, professional drivers, airline pilots, radar operators, and in many other fields where single observers are relied on for life-altering perceptual decisions.

## Data availability statement

The original contributions presented in the study are included in the article/supplementary material, further inquiries can be directed to the corresponding author.

## Ethics statement

The studies involving human participants were reviewed and approved by the Committee for the Protection of Human Subjects at the University of California, Berkeley. The patients/participants provided their written informed consent to participate in this study.

## Author contributions

ZW, MM, ZR, and CG programmed the software. MM, ZR, CG, and MZ performed the data collection. ZW, MM, ZR, and TC-B programmed the pipeline to perform the data analysis. ZW and DW drafted the manuscript. MM, YM, and DW reviewed and edited the manuscript. MM and ZW made the figures. All authors contributed to the article and approved the submitted version.

## Funding

This work was supported in part by the National Institutes of Health (grant number: R01 CA236793-01). Publication made possible in part by support from the Berkeley Research Impact Initiative (BRII) sponsored by the UC Berkeley Library.

## Conflict of interest

The authors declare that the research was conducted in the absence of any commercial or financial relationships that could be construed as a potential conflict of interest.

## Publisher’s note

All claims expressed in this article are solely those of the authors and do not necessarily represent those of their affiliated organizations, or those of the publisher, the editors and the reviewers. Any product that may be evaluated in this article, or claim that may be made by its manufacturer, is not guaranteed or endorsed by the publisher.

## Footnotes

^1^Note that here *sensitivity* refers to perceptual discriminability (i.e., *d’* or Just Noticeable Difference, JND), but we acknowledge that in the medical research literature, the term "*sensitivity*" could refer to accuracy or the hit rate (independent of false alarms). For clarity and comparison to previous research, we use *sensitivity* to refer to perceptual discriminability (e.g., *d’*).
